# Supplementary material for: DA7R: A 7-Letter Zip Code to Target PDAC
Source: Pharmaceutics. 2023 May 16;15(5):1508. doi: 10.3390/pharmaceutics15051508 (PMC10221132; doi:10.3390/pharmaceutics15051508)

DA7R: A 7-Letter Zip Code to Target PDAC

Sofia Parrasia[1], Andrea Rossa[2], Nicola Roncaglia[2,3], Andrea Mattarei[4], Claudia Honisch[3], Ildikò Szabò[1], Paolo Ruzza[3]* and Lucia Biasutto[5]*

[1] Department of Biology, University of Padova, Viale G. Colombo 3, 35131 Padova, Italy;

[2] Department of Chemical Sciences, University of Padova, Via F. Marzolo 1, 35131 Padova, Italy;

[3] CNR Institute of Biomolecular Chemistry, Padua Unit, Via F. Marzolo 1, 35131 Padova, Italy;

[4] Department of Pharmaceutical and Pharmacological Sciences, University of Padova, Via F. Marzolo 5, 35131 Padova, Italy;

[5] CNR Neuroscience Institute, Padua Unit, Viale G. Colombo 3, 35131 Padova, Italy.

*Correspondence: paolo.ruzza@cnr.it; lucia.biasutto@cnr.it

SUPPLEMENTARY DATA

***Supplementary figure S1**. ESI-MS spectra (positive ion mode) of PAPTPL-Teg-N_3, FITC-DA7R, PAPTPL-I-DA7R, PAPTPL-Teg-DA7R and PAPTPL-Teg-cA7R.*

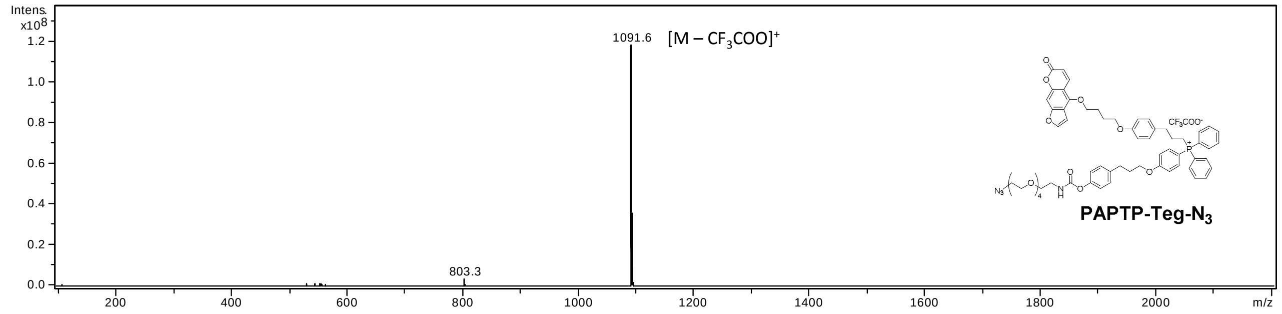

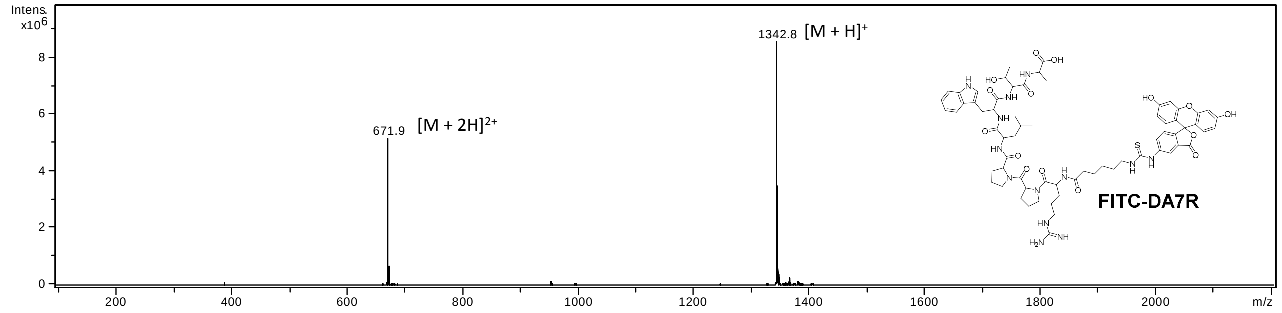

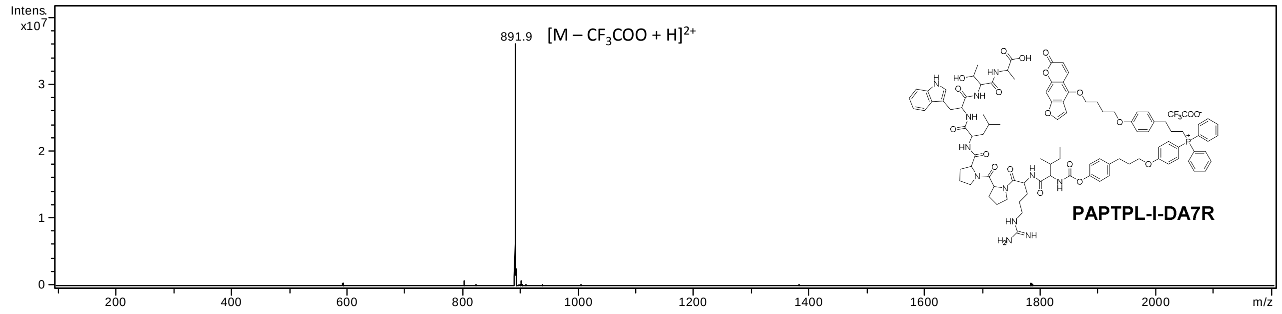

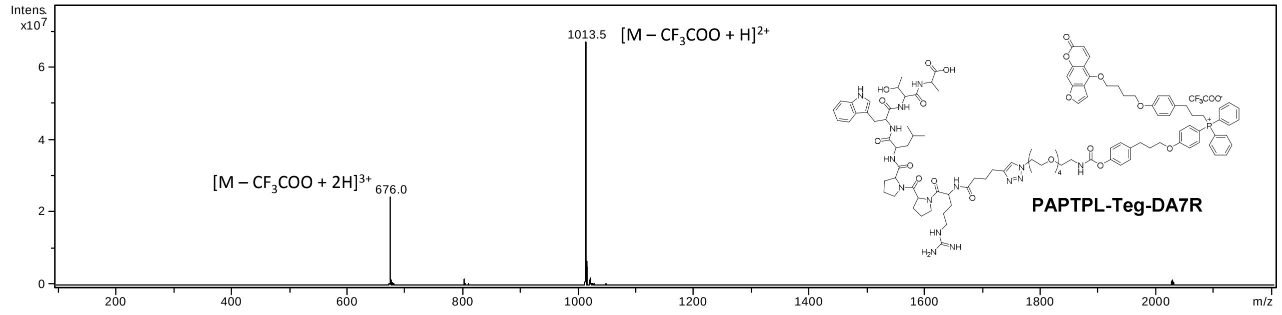

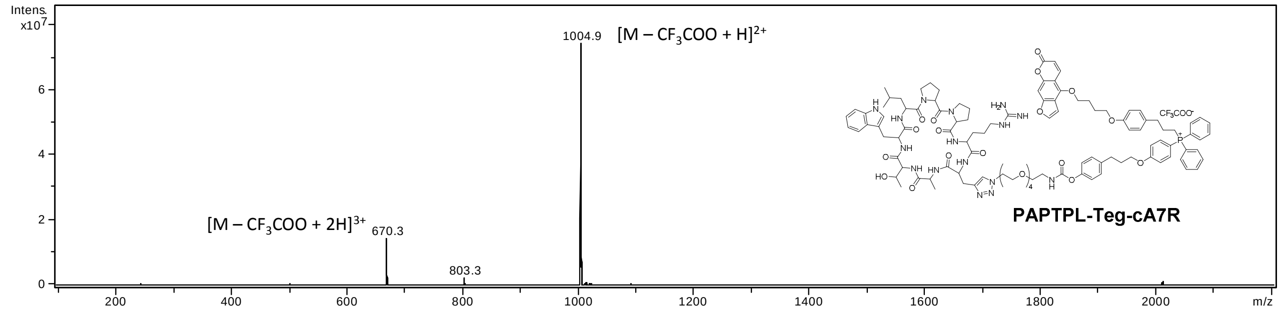

Supplementary figure S2. *^{1}H-NMR spectrum of 4-nitrophenyl (14-azido-3,6,9,12-tetraoxatetradecyl)carbamate (**1**).*

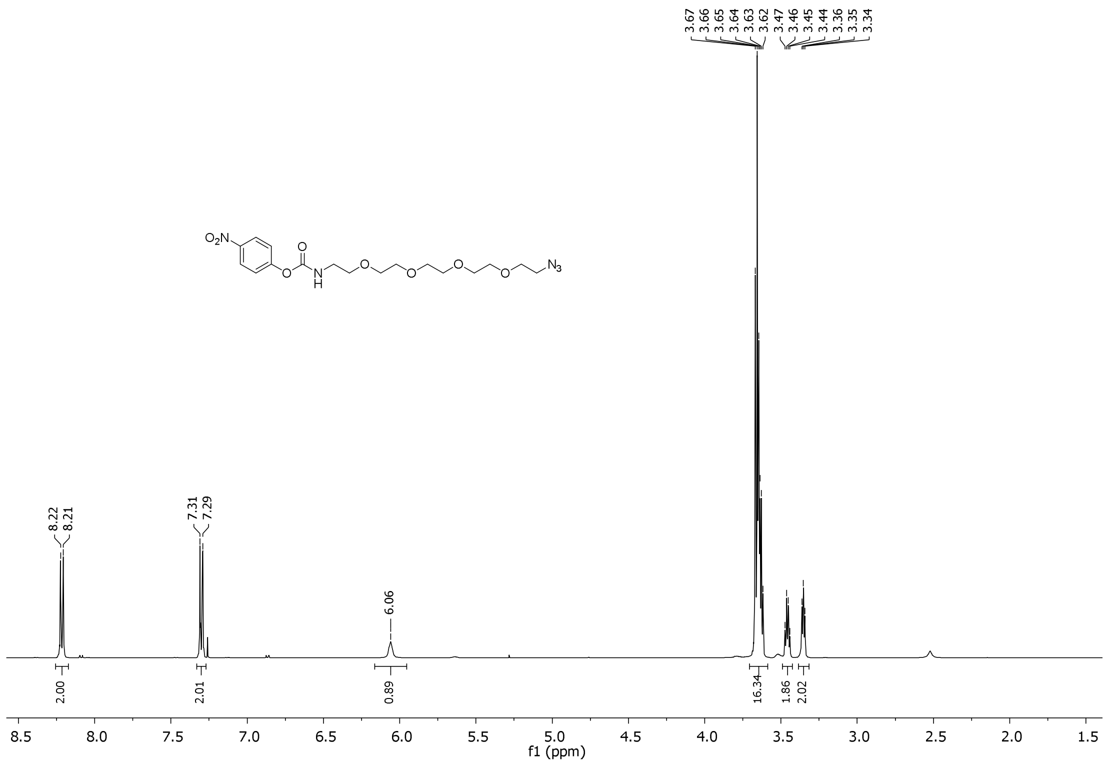

Supplementary figure S3. *^{13}C-NMR spectrum of 4-nitrophenyl (14-azido-3,6,9,12-tetraoxatetradecyl)carbamate (**1**).*

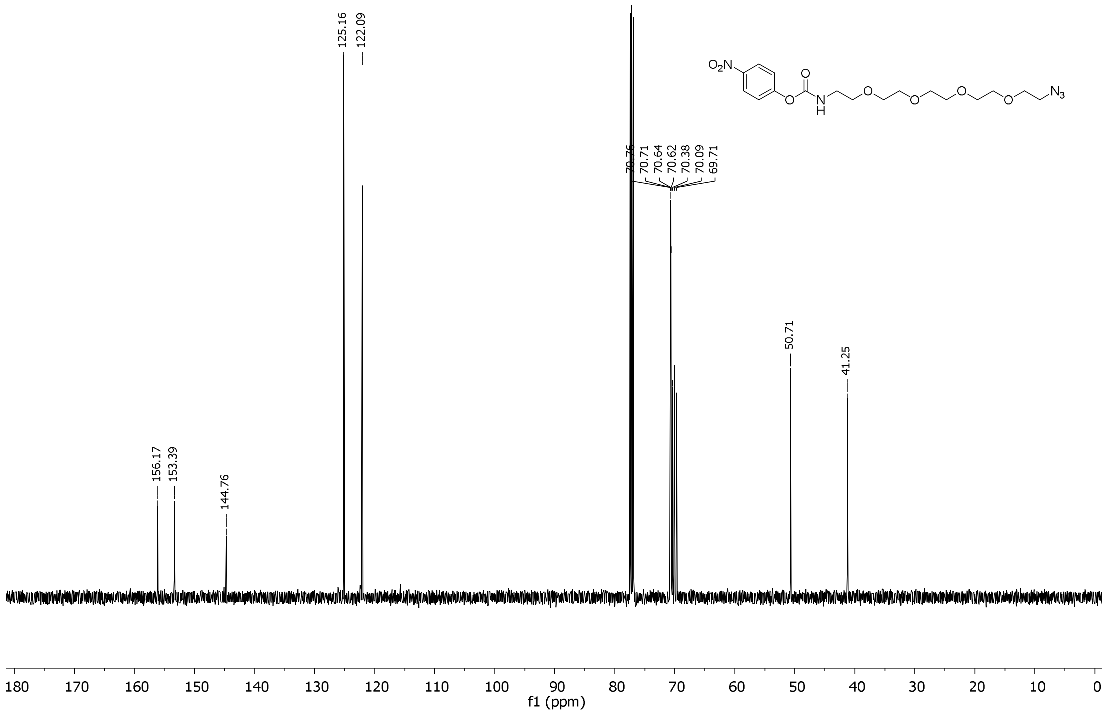

Supplementary figure S4. ^{1}H-NMR spectrum of PAPTPL-Teg-N_3.

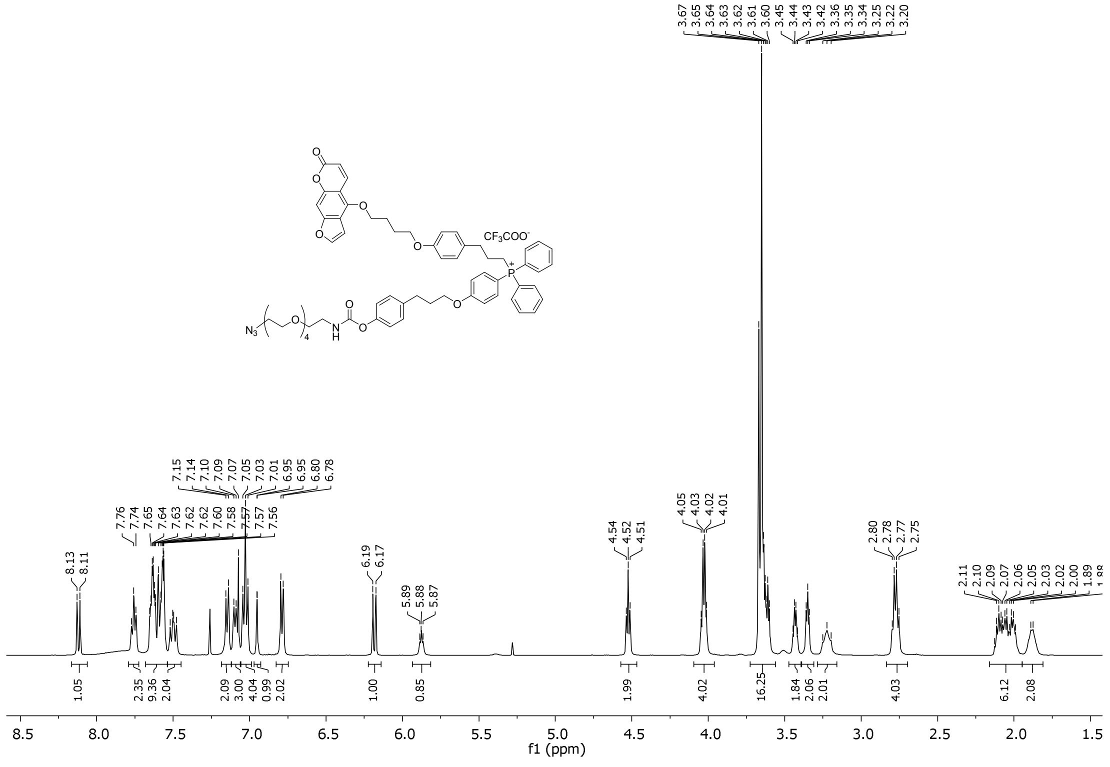

Supplementary figure S5. ^{13}C-NMR spectrum of PAPTPL-Teg-N_3.

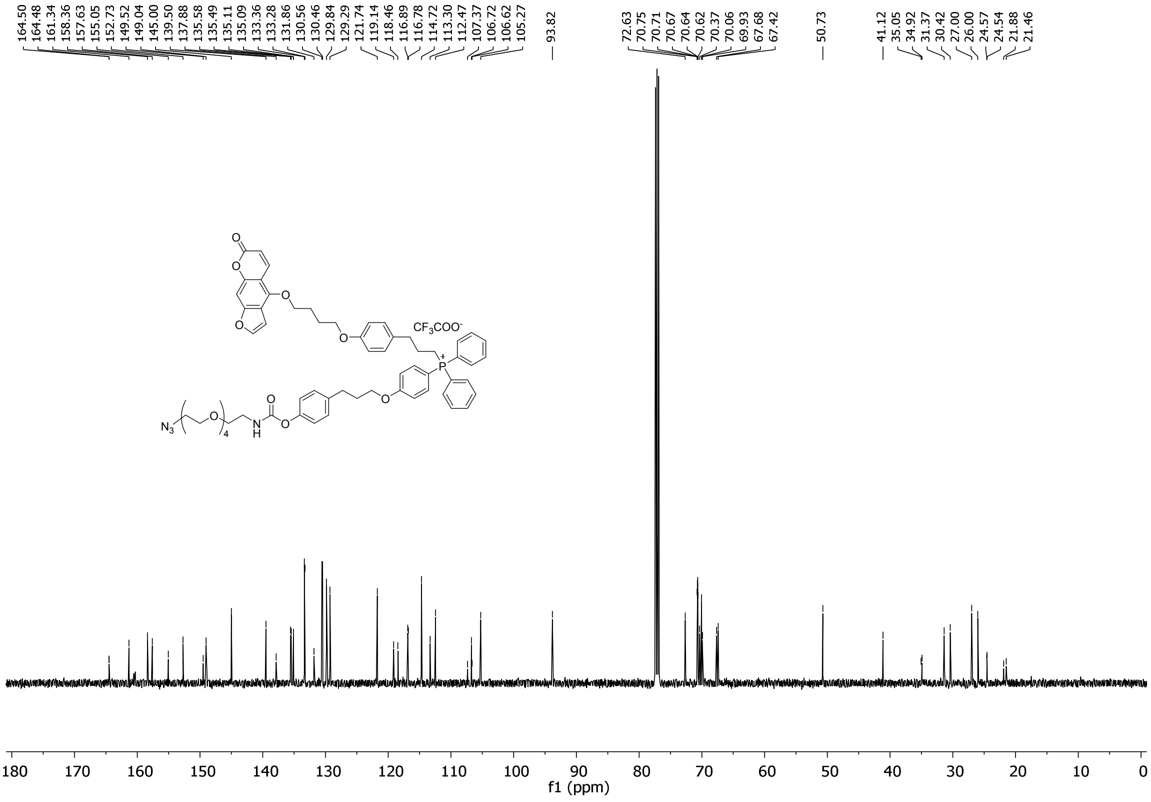

Supplementary figure S6. *HPLC/UV chromatograms (312 nm) of PAPTPL-I-DA7R (A), PAPTPL-Teg-DA7R (B) and PAPTPL-Teg-cA7R (C).*

A

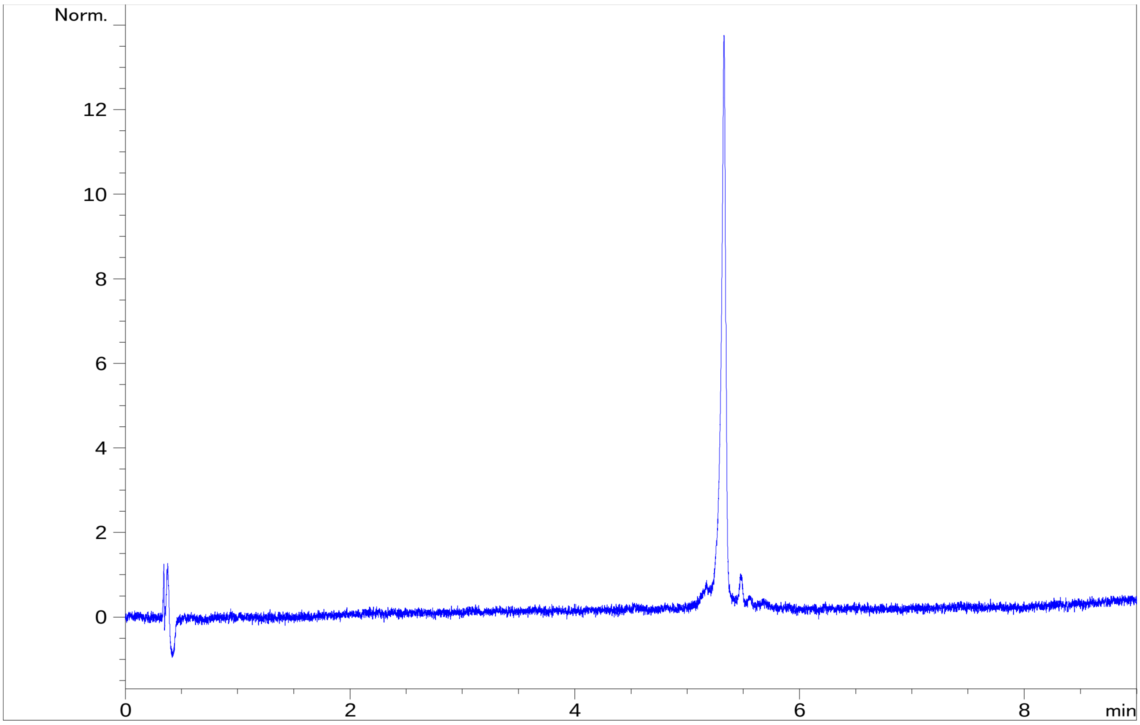

B

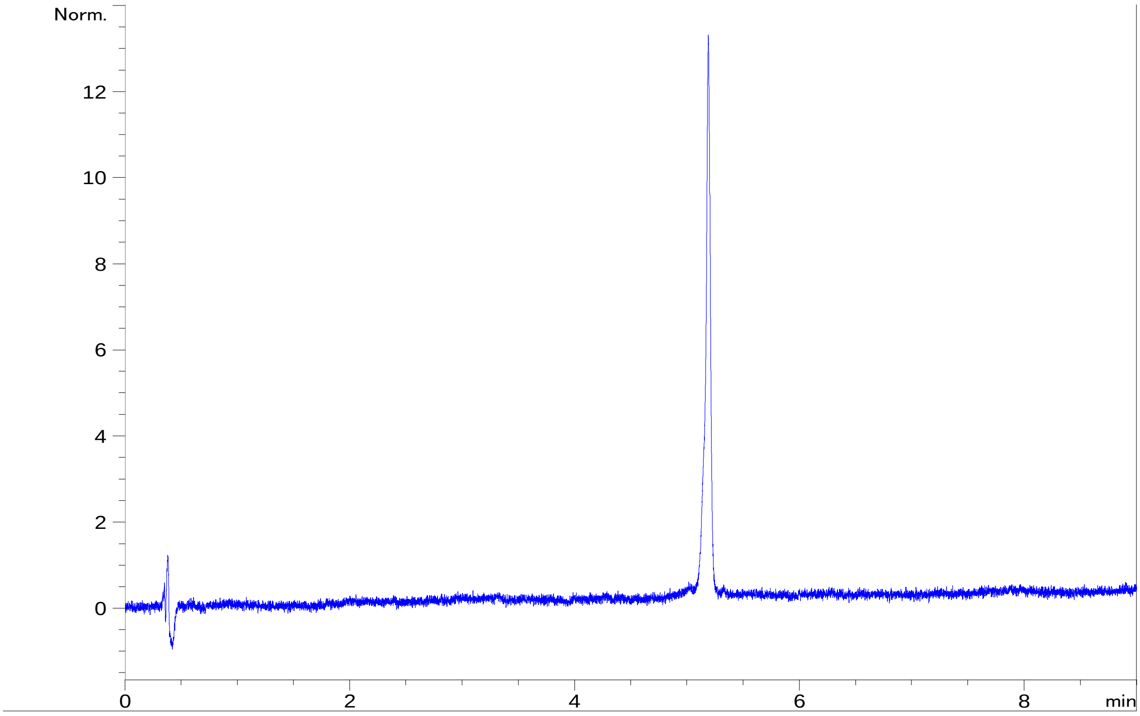

C

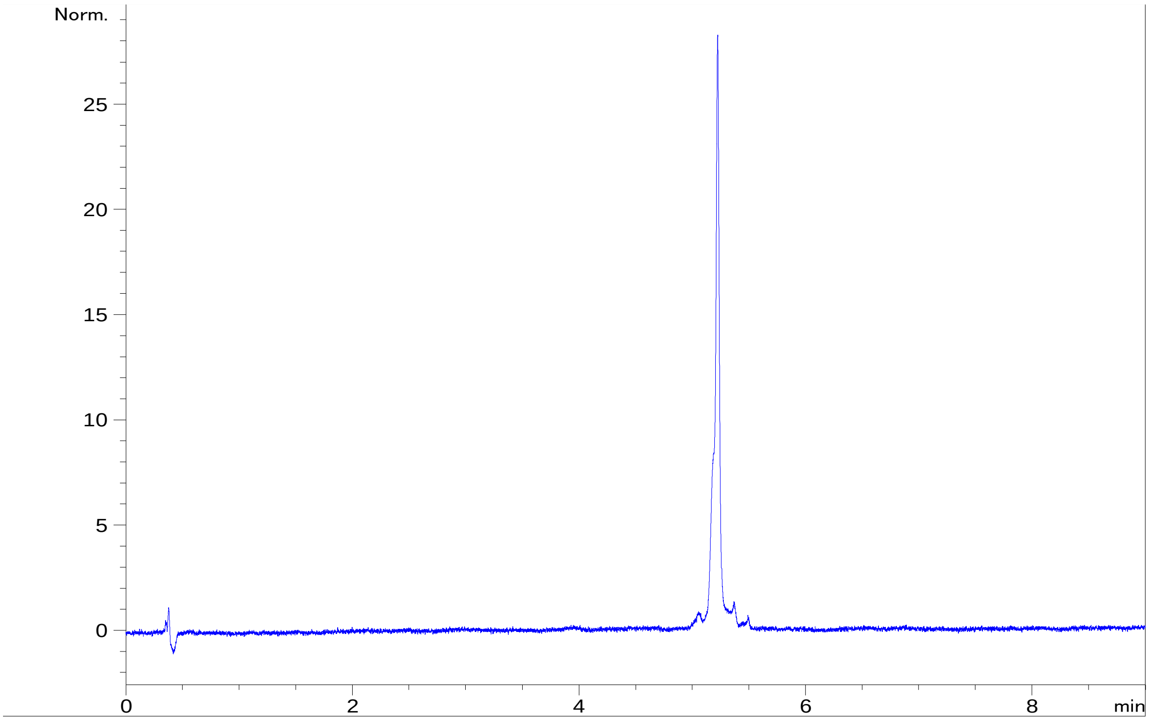

Supplementary Figure S7. *Representative Western Blot image of total protein levels (Pounceau staining) in lysates from different PDAC cells lines. Each lane was loaded with 30 µg of proteins.*

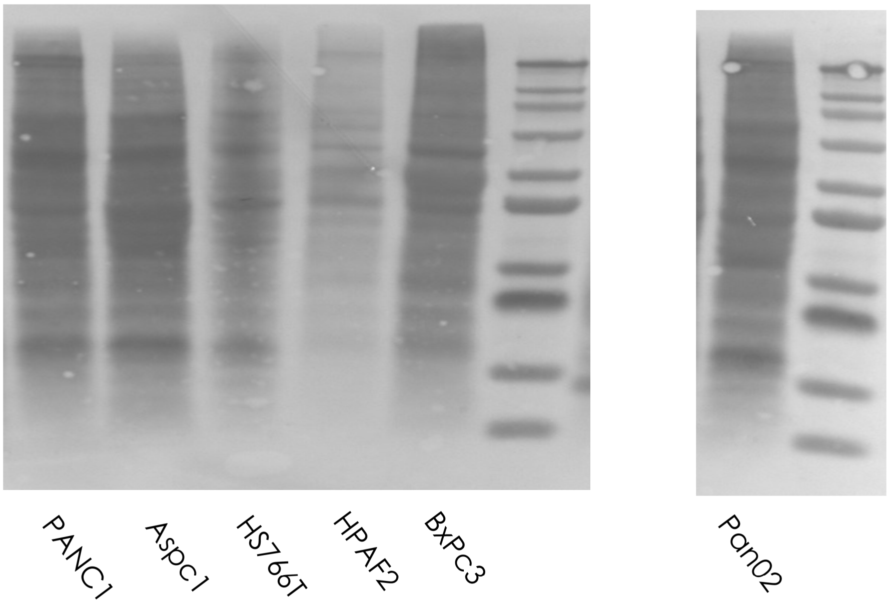

Supplement: Supplementary file 1 [file pharmaceutics-15-01508-s001.zip › pharmaceutics-2361190-supplementary.pdf]
